# Powder for Wounds

**Published:** 1898-09

**Authors:** 


Powder for Wounds. A mixture consisting of equal parts of iodoform and calomel is an excellent application for recent wounds, etc.

